# Computationally Informed Design of a Multi-Axial Actuated Microfluidic Chip Device

**DOI:** 10.1038/s41598-017-05237-9

**Published:** 2017-07-14

**Authors:** Alessio Gizzi, Sara Maria Giannitelli, Marcella Trombetta, Christian Cherubini, Simonetta Filippi, Adele De Ninno, Luca Businaro, Annamaria Gerardino, Alberto Rainer

**Affiliations:** 1 Università Campus Bio-Medico di Roma, Department of Engineering, Rome, Italy; 2 International Center for Relativistic Astrophysics (ICRA), Rome, Italy; 3 Institute for Photonics and Nanotechnology, National Research Council, Rome, Italy

## Abstract

This paper describes the computationally informed design and experimental validation of a microfluidic chip device with multi-axial stretching capabilities. The device, based on PDMS soft-lithography, consisted of a thin porous membrane, mounted between two fluidic compartments, and tensioned via a set of vacuum-driven actuators. A finite element analysis solver implementing a set of different nonlinear elastic and hyperelastic material models was used to drive the design and optimization of chip geometry and to investigate the resulting deformation patterns under multi-axial loading. Computational results were cross-validated by experimental testing of prototypal devices featuring the *in silico* optimized geometry. The proposed methodology represents a suite of computationally handy simulation tools that might find application in the design and *in silico* mechanical characterization of a wide range of stretchable microfluidic devices.

## Introduction

Notable technological improvements of microfluidic devices have been conducted over the last decade^1–6^ bringing lab-on-a-chip^7–13^ and organ-on-a-chip applications^14–17^ to the mainstream. High throughput screening represents the major outcome of such a vast technological improvement that necessitates a fine control over microfluidic, mechanical, and multiphysical interactions^18^. Mechanotransduction and mechanosensitivity are universally recognized as fundamental pathways for the correct physiological development of *in vitro* tissues^19, 20^, and this aspect has been long investigated in tissue-engineered models by applying mechanical stimulation to substrates or scaffolds seeded with cells^21, 22^. However, with the advances in micro- and nano-scale technologies, a new class of microfabricated devices for the study of biological processes under mechanical stimulation^18, 23^ has been developed. In this framework, the notable work from groups as the one led by D.E. Ingber has posed the basis for obtaining microfluidic platforms integrating mechanical stretching and fluid flow conditions^24–26^ to successfully recapitulate human disease models on a chip. However, most examples in the literature focused on uniaxial stretching, and the few examples of multi-axial devices^23, 27^ did not unveil the potential of fully programmable actuation along different directions. In all cases, comprehensive theoretical modeling and engineering optimization of the mechanically actuated devices are limited to specific applications^28^. The complex interaction between cells and substrates has been recently investigated through advanced theoretical and computational modeling approaches^29–33^, further highlighting the tight interplay of different multiphysical effects during mechanotransduction processes, i.e. fluid-electro-mechanics. These studies, based on *in vitro* evidences, indicated a clear route for future microfluidic technologies: optimization of the environmental constraints on on-chip cell cultures mimicking *in vivo* conditions in a more reliable way.

In the present contribution, we propose a novel vacuum-actuated multi-axial microfluidic chip device (MCD) obtained as the result of mathematical modeling, computationally informed design, and optimization strategies in closed loop with microfabrication processes and experimental analyses. We based our numerical model and *in silico* analyses on a solid theoretical description of the materials undergoing mechanical deformation in the large strain regime. Numerical analyses were conducted via parametric optimization of the MCD structural features with the aim to tailor the induced multi-axial deformation field. The computational model of the MCD was finally validated against experimental evidences on a prototypal device. The joint theoretical, numerical, and experimental work results in a new reliable toolbox for optimized lab-on-a-chip applications.

## Results

### Structural elements of a multi-axial stretchable MCD

**Figure 1:**
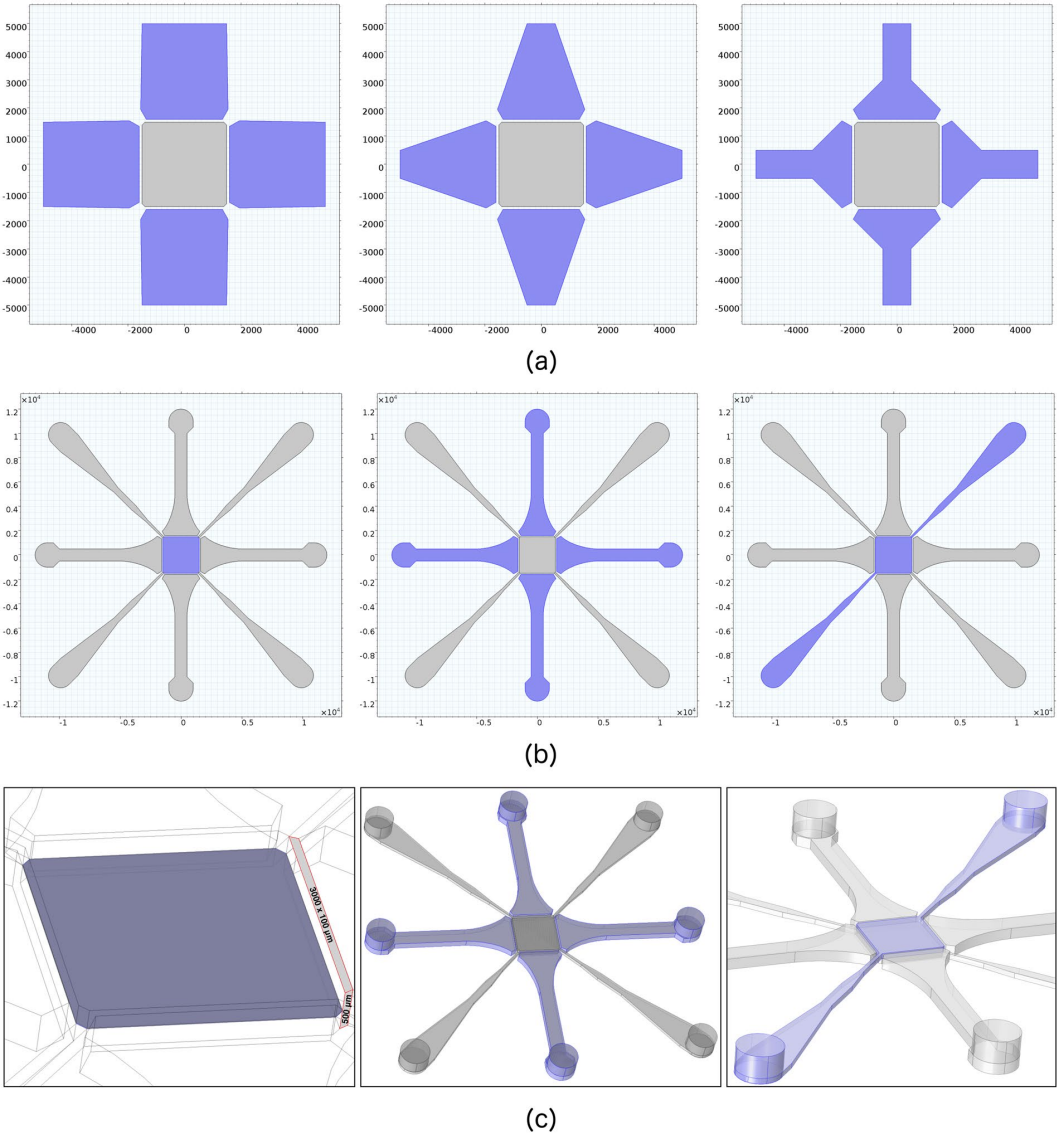
Key structural elements of the MCD. (**a**) Planar view of three different shape configurations for the vacuum actuators (in blue). (**b,c**) Planar view (**b**) and three-dimensional sketch (**c**) of the MCD structure. From left to right, the porous membrane (PM), the vacuum chambers (VC), and the perfusion channels (PC) are highlighted in blue colour. Length scale in [*μm*].

The MCD is based on elastomeric polydimethylsiloxane (PDMS), and its main components consist of a porous membrane (PM), actuated by four vacuum chambers (VC) at its sides, and perfused through a set of perfusion channels (PC). A careful *in silico* design optimisation process was performed with particular reference to the shape of the PM and of the VCs. Details about the different configurations analysed are provided in supplementary information (see Figs S1 and S2). In brief, three optimum criteria were adopted: i) maximisation of the strain field induced on the PM for the maximum applicable load; ii) control and distribution of the strain field maximising the PM surface usage; and iii) minimisation of the bending stiffness of the VC walls at the interface with the PM. Although the selected optimum requirements may be easily derived for single structural elements, i.e. the PM and the VC walls, their coupling within a three-dimensional device with features at different length scales requires the support of computational tools described in detail in the next section. Figure 1(a) shows a representative example of three different VC geometries that were analysed. Each configuration was characterised in terms of the three mentioned optimum criteria. In particular, from left to right, the maximum strain level induced on the PM increases due to the minimisation of the stiffness at the membrane-wall interface. Moreover, the strain gradient decreases, producing a more uniform strain field on the PM.

The resulting optimised geometry of the MCD is shown in Fig. 1(b,c), with its key structural elements evidenced in blue colour. The optimised MCD features a 3 × 3 *mm* ^2^ culture chamber bordered with a flexible wall with a cross-sectional size of 100 *μm* (width) × 500 *μm* (height). The PM is a planar squared thin structural element clamped along its edges at the VC walls. The PM does not provide any bending stiffness and is modelled as a plane stress element in 3D with the possibility to deform both in the in-plane and out-of-plane directions. The PM provides the structural support for cells under controllable strain fields and it further allows fluids to diffuse between the upper and lower culture chambers. The VCs are designed as elongated *chess pawns* arranged in a cross fashion along the edges of the PM (Fig. 1(a)). The chosen shape provides two salient features to the device: i) it allows to minimise the stiffness of the VC walls at the interface with the PM, thus maximising the entity of PM stretching; ii) it avoids potential collapse of the VCs undergoing negative pressure. In addition, VCs are not communicating and hence can be controlled independently, allowing true multi-axial actuation to be performed on the PM. Two pairs of inlet-outlet PCs selectively control the fluidics of the upper and lower culture chambers. Insertion of the PCs occurs at the chamber corners, and their width is designed to trade off between perfusion efficiency and constraint to PM actuation.

**Figure 2:**
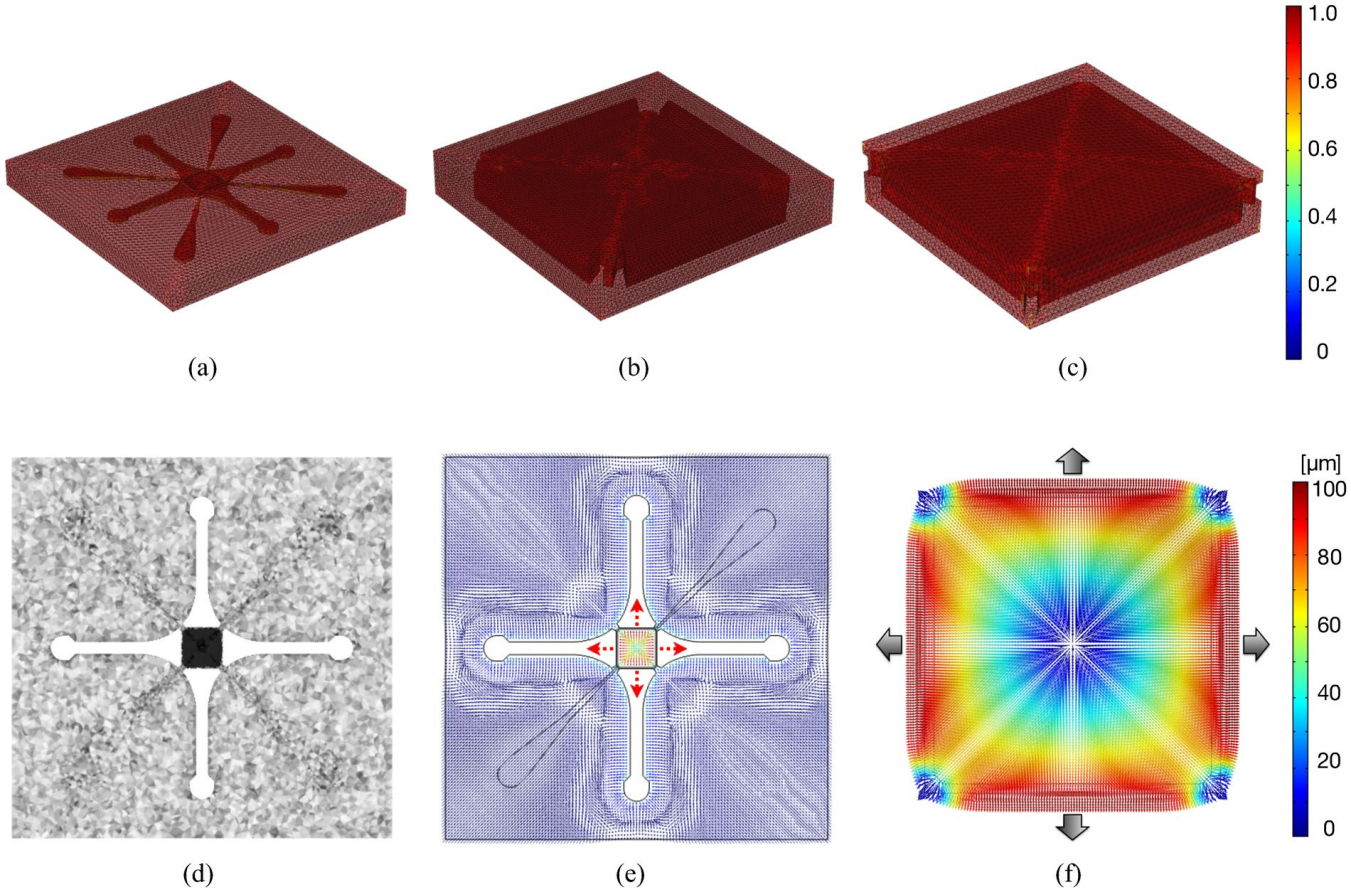
Computational model. (**a–c**) Mesh element quality distribution over the entire MCD (a) and two progressive zoomed views of the culture chamber (**b,c**). Colour code refers to tetrahedral mesh quality (1 represents the highest quality). (**d,e**) Mesh distribution (**d**) and arrow plot of the displacement field (**e**) along the mid-planar section for the entire device. (**f**) Zoomed view on the displacement field on the PM (the equibiaxial loading case is highlighted by pressure arrows).

A representative example of the spatial discretisation quality adopted for numerical analysis is shown in Fig. 2 (a view of the whole device and two zoomed views of the PM are reported (a-c)). The bulk region of the MCD was discretised by using tetrahedral elements with maximum size of 80 *μm* and an advancing front meshing protocol was used to discretise the connected membrane with minimum element size of 40 *μm* (Fig. 2(d)). Such a discretisation allowed us to provide a sufficient number of finite elements between the PM and the device external boundaries, avoiding non-realistic membrane deflections, and correctly solving both the nonlinear elastic and the hyperelastic problems. The final optimised simulation setup, reduced to the sole region surrounding the PM, consisted of $\sim 6\cdot {10}^{4}$ elements, corresponding to $\sim 3.3\cdot {10}^{5}$ degrees of freedom. Such an optimised computational model required about 8 GB of RAM, achieving the purposed target of modest computational effort. High performance computing analysis was also performed on the whole MCD domain: numerical solution consisted of $\sim 1.5\cdot {10}^{6}$ d.o.f. and $\sim 3\cdot {10}^{5}$ elements. Negligible discrepancies were observed between the reduced and the full MCD models. In both cases (i.e., reduced and full MCD geometry), computational time and cost were not substantially affected by the specific nonlinear elastic or hyperelastic material model chosen for the solution. The displacement field over the mid-planar section of the whole MCD is shown in Fig. 2(e) for an equibiaxial loading with *p* = −500 *mbar*. The normalized arrow plot highlights the expected symmetry of the solution. Figure 2(f) shows a zoom of the displacement arrow plot on the membrane surface. As expected, the applied negative pressure induced notable displacements (up to $\sim 100\,\mu m$) on the sole PM element.

### MCD physics is compatible with multiple stretching regimes

**Figure 3:**
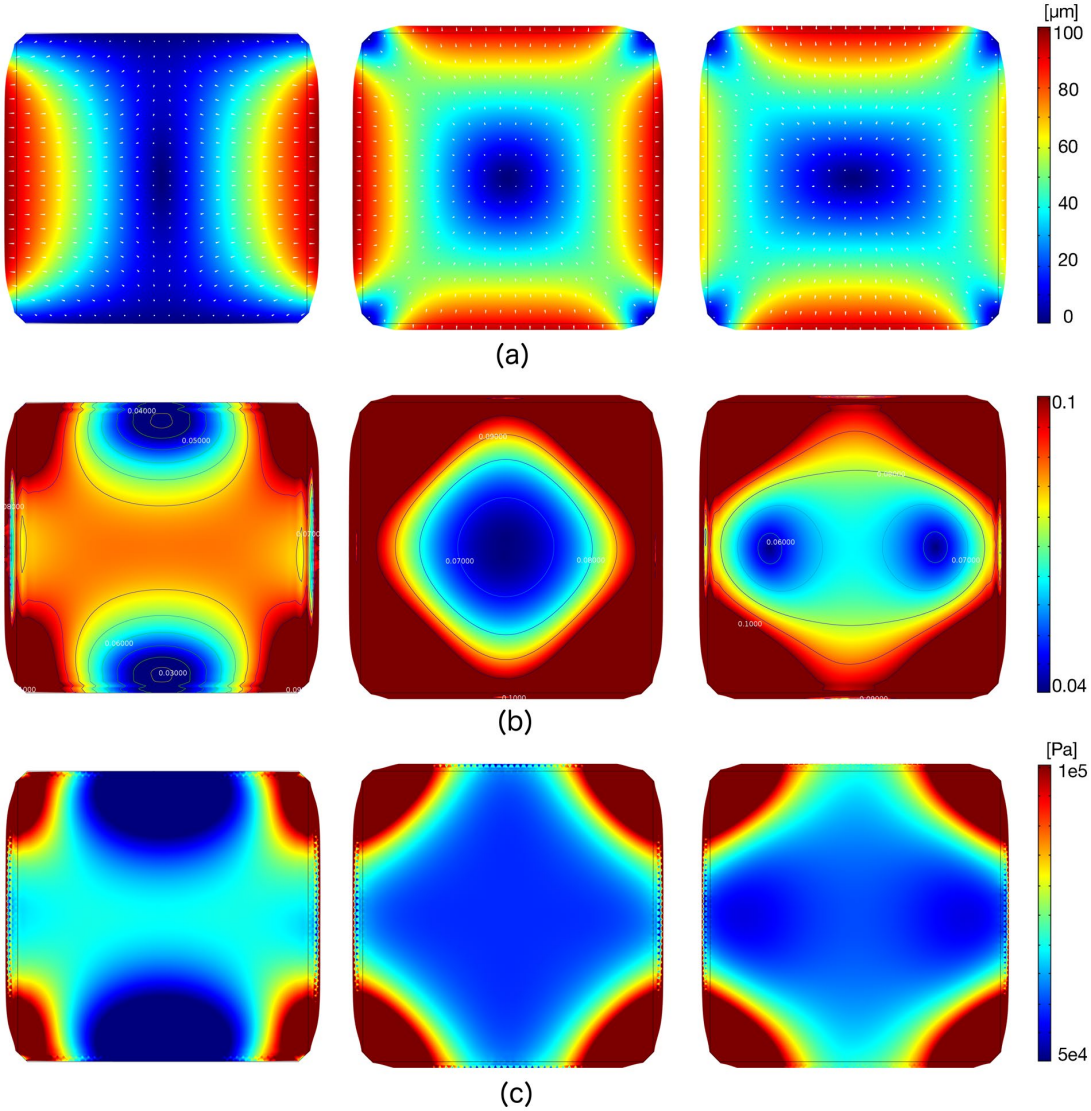
Results of numerical analysis. (**a**) Displacement field induced on the PM under uniaxial (left), equibiaxial (center) and biaxial 3:5 (right) loading patterns for a maximum pressure *p* = −500 *mbar*. White arrows indicate the local horizontal and vertical components of the displacement field. (**b**) Color map and isolevel contours of the first invariant of deformation for the corresponding loading patterns. A limited range of strain values is displayed for the three cases, i.e. [0.04 ÷ 0.1]. (**c**) Color map of the von Mises stress distribution for the three loading patterns. A limited range of stress levels ([0.5 ÷ 1] · 10^5^ *Pa*) is displayed.

In order to appreciate the accurate control over the displacement/strain fields of the membrane structure, we describe the results of the multiple numerical analyses conducted with particular attention to the PM response. Figure 3 shows the displacement field (Fig. 3(a)), the strain map (first invariant of the deformation, Fig. 3(b)), and the von Mises stress (Fig. 3(c)) obtained on the PM for three different loading patterns: i) uniaxial loading with *p* = −500 *mbar* (left); ii) equibiaxial loading with *p* = −500 *mbar* (center); iii) biaxial loading with *p*_1_ = −300 *mbar* and *p*_2_ = −500 *mbar* (right). For each simulated loading pattern, the maximum value of the displacement ($\sim 100\,\mu m$) was obtained on the boundaries of the membrane corresponding with the loaded surfaces. Results confirmed the strong differences between the three cases and emphasized some notable features of the MCD. As expected, uniaxial loading produced an almost mono-axial displacement field in the direction of the applied load covering a large portion of the PM surface, with minor deviations on edges connected to non-loaded walls. The equibiaxial case showed a highly symmetric solution with a large central portion of the PM exhibiting a radially oriented displacement field. We remark the close analogy with the analytical solution of the plane strain problem. The biaxial loading case—which can be interpreted to some extent as the superposition of uniaxial and equibiaxial loadings—showed an expected displacement field, with a complex associated strain pattern, characterized by two localized regions of minimum strain within the center portion of the PM. In all cases, we noticed singularities in the displacement field at the PM corners in proximity to the PC insertions, acting as fixed constraints to PM stretching.

### Adherence of *in silico* model to experimental evidence

**Figure 4:**
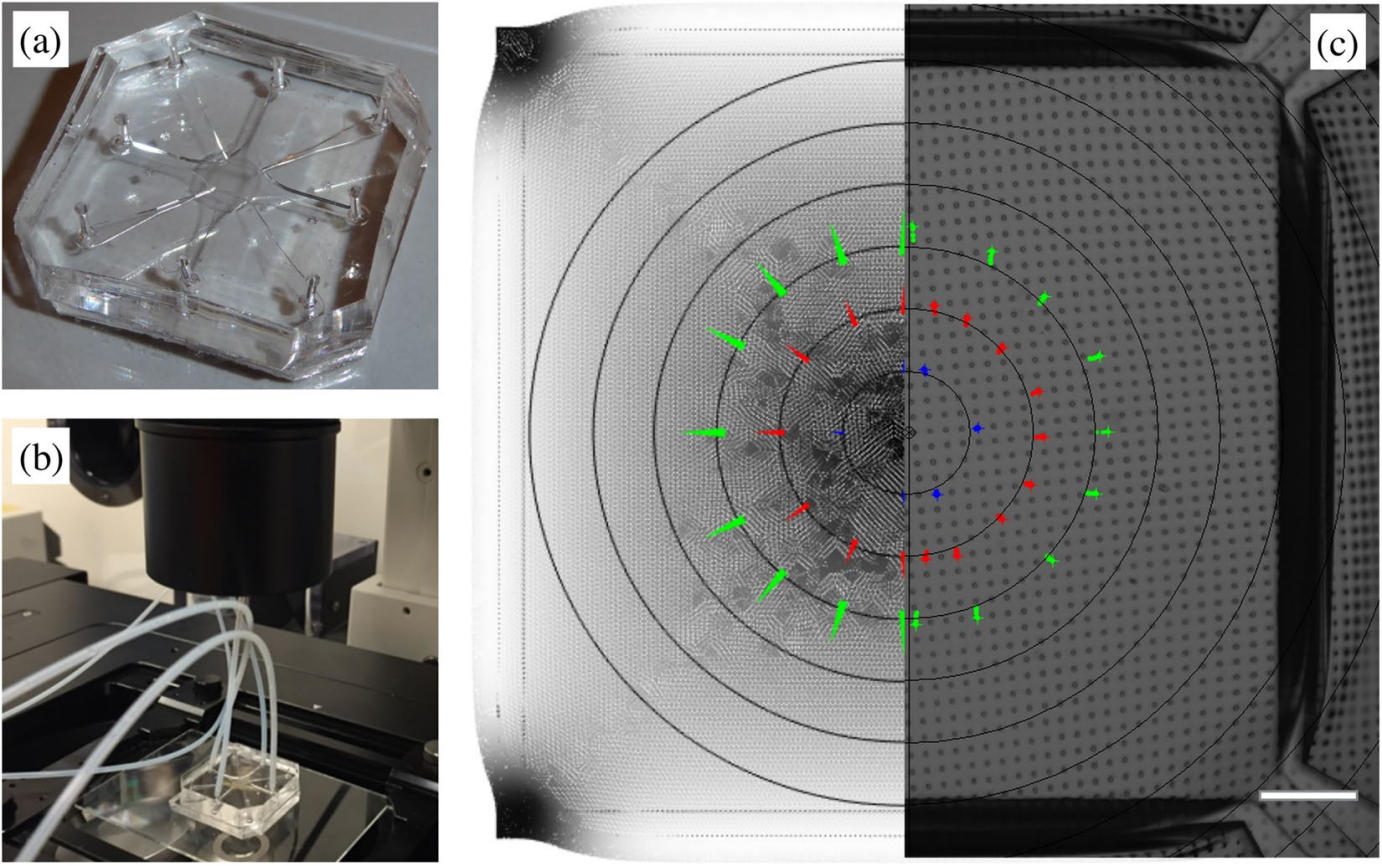
MCD actuation. (**a,b**) Optical macrographs of the MCD prototype (**a**) and of the actuation setup (**b**). (**c**) 1:1 comparison between simulated (left half) and experimental (right half) displacement fields for the porous membrane (PM) under actuation at a vacuum level of – 500 *mbar* (scale bar: 200 *μm*). Arrows in color highlight displacement vectors for a set of markers at different distances (250, 500, 750 *μm*) from the center of the membrane.

**Figure 5:**
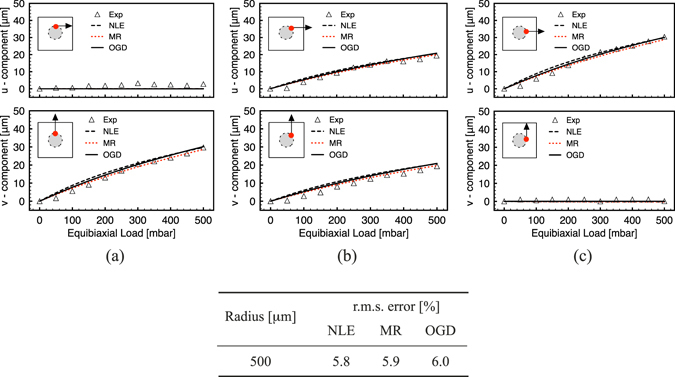
Model validation under equibiaxial loading (negative pressure). (**a–c**) Displacement field components (horizontal and vertical, *u*, *v*, respectively) taken at *r* = 500 *μm* from the center of the PM for three representative points. ‘Exp’ refers to measured data as the mean of three independent experiments on different devices; NLE, MR and OGD refer to nonlinear elastic (7), Moonery-Rivlin (8) and Ogden (9) material models, respectively. The insets indicate the position of the points with coordinates (origin is set in the center of the membrane): (**a**) (0, 500), (**b**) (353, 353), (**c**) (500, 0). The table reports the average percentage error of the displacement for the three selected points for the peak pressure (500 *mbar*) vs. the three material models.

Following computational analysis, the optimized MCD geometry was translated into a prototypal device fabricated by PDMS soft-lithography (see Fig. 4(a)). We conclude our analysis describing the model validation procedure and highlighting its statistical significance with respect to measurements performed on the real device (see Fig. 4(b,c)). A representative comparison is provided in the following and an extended set of tracking results is reported in Supplementary Fig. S4. Figure 5 compares the equibiaxial displacement field components (*u*, *v* horizontal and vertical, respectively) measured on the MCD with those obtained by numerical simulations for the three material models (see Eqs (7)–(9) in the Methods section). Three different points placed at (0°, 45°, 90°) along a circular region with radius 500 *μm* from the center of the PM have been represented. Measurements performed on these points confirmed the expected displacement pattern and closely matched the *in silico* data up to the highest loading pressure (radial orientation of the displacement field with a modulus of $\sim 30\,\mu m$).

The inset table in Fig. 5 reports the r.m.s. error between the experimentally measured and the *in silico* data for the three different material models. It is worth noting that all the models provided a similar reliability, with a maximum error of 6% for the Ogden material model (9). Major deviations with respect to the measured data could be evidenced at small loadings and along the diagonal path. These were due to singularity displacement lines (see Fig. 3(a) (center)) ending on the PCs that constrained the PM stretching. In fact, as highlighted in Fig. 3(b) (center), the isolevels of the first invariant of strain deviated from the circular analytical solution and presented a higher strain gradient along with the diagonal paths ending on the PCs. Accordingly, also the stress isolevels presented similar features (see Fig. 3(c) (center)). Similar validation analyses were also performed under uniaxial loadings providing the same qualitative and quantitative results.

## Discussion

The above reported results underline the modularity and adaptability of our MCD to provide truly multi-axial strain states toward multiple and diverse mechanobiology applications. This device would give the possibility not only to modulate the intensity of cell culture experienced stretching over time, but also to modify its spatial distribution—according to a priori established patterns—mimicking, e.g., the insurgence of pathologic conditions on *in vitro* tissue models. We refer to Huh *et al*.^34^ for an extended review on organ-on-a-chip models recapitulating the physical microenvironment of healthy and injured organs.

When analysing the behavior of the PM, it is worth noting that, being PDMS a nonlinear material, local membrane stiffness might depend upon punctual strain level. This might have important implications in a device destined for cell culture, given the key role of substrate stiffness in driving cellular responses^35^. Hence, the spatial distribution of the equivalent material stiffness on the PM domain was also included in the computational analysis. In our settings, the center portion of the PM remained in the pseudo-linear regime, with an almost constant elastic modulus in the 5 · 10^5^ *kPa* range regardless the spatial distribution of the strain field (see Supplementary Fig. S3).

In the perspective of further optimizing the computational toolbox, two main factors shall be mentioned: (1) the computational model does not consider any pre-stretch on the PM (that cannot be estimated a priori in our experimental setup); (2) the CAD geometry implemented in the numerical code does not account for micro-scale geometric inaccuracies introduced during alignment and plasma-bonding of the MCD layers. These aspects would require a massive statistical analysis of multiple devices and their testing under different working conditions. In addition, the mechanical characterization of the MCD encompasses microstructural formulation and micromechanical interface simulations^36–41^ toward the study of the growth and remodeling of cultured cell layers within a microfluidic actuated environment. These will be addressed by introducing multiphysics coupling at the cell and tissue level—e.g., viscosity^42^, electro-mechanics^43^ and fluid-structure interaction^44^—with the final aim to provide a comprehensive computational tool. A dedicated study will finally extend the theoretical description of the PM by adopting micropolar and second gradient homogenization techniques dedicated to media with periodic microstructures^45–48^. This extension, in particular, will allow us to characterize nonlocal effects^49^ and to incorporate multiscale feedbacks at the cellular level^30^. Accordingly, advanced theoretical description of nonlinear diffusion in soft porous media^50^ will allow the characterization of multiphysical emergent behaviors on the basis of a sound thermodynamic framework^51, 52^.

## Methods

### Constitutive Modeling of the Microfluidic Device

PDMS is an isotropic, incompressible, and stress-asymmetric material, which shows a strong dependance of its nonlinear mechanical properties upon the pre-polymer to catalyst ratio. We chose two different compositions (i.e., 15:1 and 10:1 v/v) in order to provide different compliance levels for the PM and the MCD main body, respectively. Mechanical behavior of PDMS at the selected compositions was evaluated via uniaxial tensile and compressive tests on dedicated specimens (see supplementary information for details). The experimentally derived PDMS properties were implemented into the computational model using both nonlinear elastic and hyperelastic material models.

In the following, according to the notation used in Holzapfel *et al*.^53^, we assume index notation for vectors and tensors and use the notation [**A**] to indicate the matrix representation of the tensor **A** in a given basis. Under finite kinematics assumptions, we define the model equations by using a general curvilinear coordinate system relating a reference (material) domain with a current (spatial) one. The coordinates in the reference domain *Ω*_0_ with boundary *∂Ω*_0_ are denoted by **X** = *X*_*I*_ (*I* = 1, 2, 3), while the current domain *Ω* with boundary *∂Ω* holds **x** = *x*_*i*_ (*i* = 1, 2, 3) and the corresponding displacement field, expressed in terms of the material coordinates, is defined as **u** = **x**(**X**) − **X**. Uppercase and lowercase subscriptions refer to the material and spatial configurations, respectively. We indicate with **F** = ∇_**X**_ **x** = *F*_*iJ*_ the two-point deformation gradient tensor and with **C** = **F** ^*T*^ **F** = *C*_*IJ*_ the right Cauchy-Green deformation tensor. We comply with the usual assumption of nearly incompressible hyperelastic materials with the strain energy density that decomposes into two terms, *Ψ* = *Ψ*_vol_ + *Ψ*_iso_. The first term, *Ψ*_vol_ = *Ψ*_vol_(*J*), accounts for volume changes, and is dependent on the volumetric deformation expressed by the Jacobian of the deformation gradient, *J* = det**F**. The second term, ${\Psi }_{{\rm{iso}}}={\Psi }_{{\rm{iso}}}({\overline{I}}_{1},{\overline{I}}_{2})$, accounts for the isochoric behavior of the isotropic constituents of the material. The isotropic term is assumed to be dependent on the first and second invariants, ${\overline{I}}_{1}$ and ${\overline{I}}_{2}$, of the modified right Cauchy-Green deformation tensor $\overline{{\bf{C}}}={\overline{{\bf{F}}}}^{T}\overline{{\bf{F}}}$, where $\overline{{\bf{F}}}={J}^{-\mathrm{1/3}}\overline{{\bf{F}}}$. The variational procedure allows us to derive the general explicit expression of the second Piola-Kirchhoff stress tensor as

1 $$\begin{array}{ccc}{\bf{S}} & = & 2\frac{{\rm{\partial }}{\rm{\Psi }}}{{\rm{\partial }}{\bf{C}}}=2{J}^{-2/3}(\frac{{\rm{\partial }}{{\rm{\Psi }}}_{{\rm{v}}{\rm{o}}{\rm{l}}}}{{\rm{\partial }}{\bar{I}}_{1}}+{\bar{I}}_{1}\frac{{\rm{\partial }}{{\rm{\Psi }}}_{{\rm{i}}{\rm{s}}{\rm{o}}}}{{\rm{\partial }}{\bar{I}}_{2}}){\bf{I}}-2{J}^{-4/3}\frac{{\rm{\partial }}{{\rm{\Psi }}}_{{\rm{i}}{\rm{s}}{\rm{o}}}}{{\rm{\partial }}{\bar{I}}_{2}}{\bf{C}}-\frac{2}{3}({\bar{I}}_{1}\frac{{\rm{\partial }}{{\rm{\Psi }}}_{{\rm{i}}{\rm{s}}{\rm{o}}}}{{\rm{\partial }}{\bar{I}}_{1}}+2{\bar{I}}_{2}\frac{{\rm{\partial }}{{\rm{\Psi }}}_{{\rm{i}}{\rm{s}}{\rm{o}}}}{{\rm{\partial }}{\bar{I}}_{2}}){{\bf{C}}}^{-1}-pJ{{\bf{C}}}^{-1},\end{array}$$

where **I** is the identity tensor and *p* is the volumetric stress representing an arbitrary hydrostatic pressure (Lagrange multiplier) controlling the incompressibility of the material and recovered from the volumetric part of the strain energy density as *p* = ∂*Ψ*_vol_/∂*J*. Accordingly, the first two-point Piola-Kirchhoff (**P**) and the Cauchy stress (*σ*) tensors derive as **P** = **FS**, and *σ* = *J* ^−1^ **FSF** ^*T*^. In particular, **P** is necessary to fit experimental data that make use of the concept of *nominal stress*, i.e., the force in the current configuration acting on the original area. Therefore, we can write the static equilibrium conditions in terms of **P** as

2 $${\rm{\nabla }}\cdot {\bf{P}}+{\rho }_{0}{\bf{b}}=0\quad {\rm{o}}{\rm{r}}\quad \frac{{\rm{\partial }}{P}_{iJ}}{{\rm{\partial }}{X}_{J}}+{\rho }_{0}{b}_{i}=0,\quad {\rm{i}}{\rm{n}}\quad {\rm{\Omega }},$$

where *ρ*_0_ represents the mass body density in the reference configuration, and *ρ*_0_ *b*_*i*_ stands for the body force vector per unit volume, which in our case is assumed to be negligible. The equation of motion (2) will be solved by imposing suitable boundary conditions reproducing the actual loading and deformation constraints applied to the device in our experimental setup (see next section). For what follows, it is useful to introduce the spectral decomposition^54, 55^ of the second Piola-Kirchhoff stress tensor by defining the stretch ratios (*λ*), associated with the principal directions of deformation, and related to strain (*ε*) via the relation *λ* = 1 + *ε*. In general, three distinct stretch ratios, *λ*_*i*_, will be defined with associated principal referential directions, **N**_*i*_, such that, by expressing the strain energy density in terms of the principal stretches, Eq. (1) assumes the form

3 $${\bf{S}}=2\sum _{i=1}^{3}\frac{\partial {\Psi }({\lambda }_{i})}{\partial {\lambda }_{i}}\frac{\partial {\lambda }_{i}}{\partial {\bf{C}}}=\sum _{i=1}^{3}{S}_{i}{{\bf{N}}}_{i}\otimes {{\bf{N}}}_{i}\,,\quad {\rm{with}}\quad {\bf{C}}=\sum _{i=1}^{3}{\lambda }_{i}^{2}{{\bf{N}}}_{i}\otimes {{\bf{N}}}_{i},$$

where the tensor product ⊗ has been used. Here, *S*_*i*_ represent the eigenvalues of **S** that assume the explicit expression

4 $${S}_{i}=2{J}^{-\mathrm{2/3}}(\frac{\partial {\Psi }_{{\rm{vol}}}}{\partial {\overline{I}}_{1}}+{\overline{I}}_{1}\frac{\partial {\Psi }_{{\rm{iso}}}}{\partial {\overline{I}}_{2}})-2{J}^{-\mathrm{4/3}}\frac{\partial {\Psi }_{{\rm{iso}}}}{\partial {\overline{I}}_{2}}{\lambda }_{i}^{2}-\frac{2}{3}({\overline{I}}_{1}\frac{\partial {\Psi }_{{\rm{iso}}}}{\partial {\overline{I}}_{1}}+2{\overline{I}}_{2}\frac{\partial {\Psi }_{{\rm{iso}}}}{\partial {\overline{I}}_{2}})\frac{1}{{\lambda }_{i}^{2}}-\frac{pJ}{{\lambda }_{i}^{2}}.$$

Considering an isotropic material under incompressibility condition (*J* = 1), the expression of a generic uniaxial loading condition, e.g., in direction *i* = 1, in terms of deformation gradient and associated Cauchy-Green deformation tensor is as follows

5 $$[{\bf{F}}]=[\begin{array}{ccc}\lambda  & 0 & 0\\ 0 & \mathrm{1/}\sqrt{\lambda } & 0\\ 0 & 0 & \mathrm{1/}\sqrt{\lambda }\end{array}],\quad [{\bf{C}}]=[\begin{array}{ccc}{\lambda }^{2} & 0 & 0\\ 0 & \mathrm{1/}\lambda  & 0\\ 0 & 0 & \mathrm{1/}\lambda \end{array}]\,,$$

with principal stretches ${\lambda }_{1}=\lambda ,\,{\lambda }_{2,3}=1/\sqrt{\lambda }$. In this case, the sole not null component of the stress is *S*_1_ and, by using *S*_2_ = *S*_3_ = 0, we can eliminate the pressure term from Eq. 4, thus obtaining the closed-form expression

6 $${S}_{1}=2(\frac{1}{\lambda }-\frac{1}{{\lambda }^{4}})(\lambda \frac{\partial {\Psi }_{{\rm{iso}}}}{\partial {\overline{I}}_{1}}+\frac{\partial {\Psi }_{{\rm{iso}}}}{\partial {\overline{I}}_{2}}),$$

with ${\overline{I}}_{1}=({\lambda }^{2}+2/\lambda )$, ${\overline{I}}_{2}=(2\lambda +1/{\lambda }^{2})$, thus deriving *P*_1_ = *λS*_1_ and *σ*_1_ = *λ* ^2^ *S*_1_.

### Material Models

On the basis of the experimental dataset deriving from uniaxial tensile/compressive testing of PDMS specimens, in the following we comply with three alternative material models characterized by a minimal set of parameters (this choice is in line with our aim of providing a computationally handy tool):

7 $${P}_{1}^{NLE}=a[{e}^{b(\lambda -1)}-{e}^{-c(\lambda -1)}]\qquad {\rm{N}}{\rm{o}}{\rm{n}}{\rm{l}}{\rm{i}}{\rm{n}}{\rm{e}}{\rm{a}}{\rm{r}}\,{\rm{E}}{\rm{l}}{\rm{a}}{\rm{s}}{\rm{t}}{\rm{i}}{\rm{c}},$$

8 $${P}_{1}^{MR}=2(1-{\lambda }^{-3})(\lambda {c}_{1}+{c}_{2})\qquad {\rm{M}}{\rm{o}}{\rm{o}}{\rm{n}}{\rm{e}}{\rm{y}}{\textstyle \text{-}}{\rm{R}}{\rm{i}}{\rm{v}}{\rm{l}}{\rm{i}}{\rm{n}},$$

9 $${P}_{1}^{OGD}=\mu ({\lambda }^{\alpha -1}-{\lambda }^{-\frac{\alpha }{2}-1})\qquad {\rm{O}}{\rm{g}}{\rm{d}}{\rm{e}}{\rm{n}}.$$

**Figure 6:**
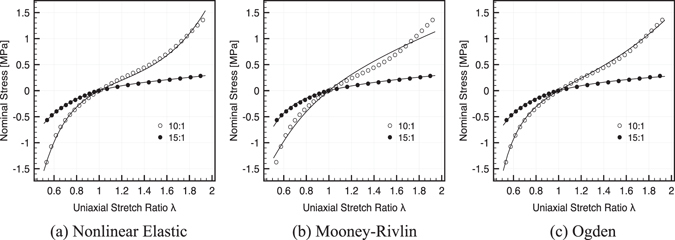
Tuning of material models. Fitting of experimental stress vs. stretch ratio curves for 10:1 v/v (empty circles) and 15:1 v/v (filled circles) PDMS using different material models: (**a**) nonlinear elastic (Eq.(7)); (**b**) hyperelastic Mooney-Rivlin (Eq. (8)); (**c**) hyperelastic Ogden (Eq. (9)). Tensile and compressive tracts are shown up to 100% and 25% strain, respectively.

Measured stress versus stretch ratio data were fit against the analytical expression provided in Eq. (7) via Matlab (The MathWorks, Inc., Natick, MA) routines by ensuring 95% of confidence, while Eqs (8) and (9) were fit via a global least-squares objective optimization algorithm making use of the Levenberg-Marquardt minimization method^56^. The optimal set of parameters is provided in Table 1 and the tensile-compressive uniaxial responses are shown in Fig. 6 for the three analytical laws and for the two PDMS compositions adopted for bulk MCD and PM structures, respectively. The resulting fit clearly shows the perfect match of the nonlinear elastic and of the hyperelastic Ogden models over the whole tensile/compressive range, with a minor deviation of the Mooney-Rivling one. PDMS shows a marked nonlinear response in the case of the 10:1 composition (bulk MCD), while the 15:1 composition (PM) has a definite linear behavior with reduced stiffness.

The obtained material characterization and model fitting is in perfect agreement with similar studies in the literature^57–60^ that analogously recognized the 2-parameter hyperelastic Ogden model as the most appropriate and computationally handy model for generalized theories of rubber-like materials.

### Numerical Analysis

For each of the three proposed material models (7–9), a computational analysis of the MCD was performed by solving the system of nonlinear partial differential eq. (2) within the finite element simulation environment COMSOL Multiphysics (COMSOL Inc., Burlington, MA) running on a multiprocessor Intel Xeon II workstation with 192 GB of RAM. Mixed cubic/quadratic Lagrange elements and different solver methods were tested to reproduce material incompressibility. Several mesh sizes, shapes and parameter tests have been implemented in order to find the optimal configuration for the numerical solution, i.e., stable numerical convergence, realistic deformation fields and negligible stress differences among comparative simulations. Boundary conditions imposed on the simulated domain correspond to the experimental set up and consisted in:

$$\begin{array}{ccc}{\bf{u}} & = & {\bf{0}}:\,{\rm{M}}{\rm{C}}{\rm{D}}{\textstyle \text{-}}{\rm{g}}{\rm{l}}{\rm{a}}{\rm{s}}{\rm{s}}\,{\rm{i}}{\rm{n}}{\rm{t}}{\rm{e}}{\rm{r}}{\rm{f}}{\rm{a}}{\rm{c}}{\rm{e}}\,({\rm{n}}{\rm{o}}\,{\rm{d}}{\rm{i}}{\rm{s}}{\rm{p}}{\rm{l}}{\rm{a}}{\rm{c}}{\rm{e}}{\rm{m}}{\rm{e}}{\rm{n}}{\rm{t}}\,{\rm{a}}{\rm{t}}\,{\rm{t}}{\rm{h}}{\rm{e}}\,{\rm{i}}{\rm{n}}{\rm{t}}{\rm{e}}{\rm{r}}{\rm{f}}{\rm{a}}{\rm{c}}{\rm{e}}\,{\rm{w}}{\rm{i}}{\rm{t}}{\rm{h}}\,{\rm{t}}{\rm{h}}{\rm{e}}\,{\rm{g}}{\rm{l}}{\rm{a}}{\rm{s}}{\rm{s}}\,{\rm{s}}{\rm{u}}{\rm{p}}{\rm{p}}{\rm{o}}{\rm{r}}{\rm{t}})\\ {\boldsymbol{\sigma }} & = & {\bf{0}}:\,{\rm{M}}{\rm{C}}{\rm{D}}{\textstyle \text{-}}{\rm{a}}{\rm{i}}{\rm{r}}\,{\rm{i}}{\rm{n}}{\rm{t}}{\rm{e}}{\rm{r}}{\rm{f}}{\rm{a}}{\rm{c}}{\rm{e}}\,({\rm{s}}{\rm{t}}{\rm{r}}{\rm{e}}{\rm{s}}{\rm{s}}{\textstyle \text{-}}{\rm{f}}{\rm{r}}{\rm{e}}{\rm{e}}\,{\rm{c}}{\rm{o}}{\rm{n}}{\rm{d}}{\rm{i}}{\rm{t}}{\rm{i}}{\rm{o}}{\rm{n}}\,{\rm{a}}{\rm{t}}\,{\rm{t}}{\rm{h}}{\rm{e}}\,{\rm{o}}{\rm{u}}{\rm{t}}{\rm{m}}{\rm{o}}{\rm{s}}{\rm{t}}\,{\rm{e}}{\rm{x}}{\rm{t}}{\rm{e}}{\rm{r}}{\rm{n}}{\rm{a}}{\rm{l}}\,{\rm{s}}{\rm{u}}{\rm{r}}{\rm{f}}{\rm{a}}{\rm{c}}{\rm{e}}{\rm{s}})\\ {\boldsymbol{\sigma }}\cdot {\bf{n}} & = & p:\,{\rm{V}}{\rm{C}}\,{\rm{s}}{\rm{u}}{\rm{r}}{\rm{f}}{\rm{a}}{\rm{c}}{\rm{e}}{\rm{s}}\,({\rm{d}}{\rm{i}}{\rm{s}}{\rm{t}}{\rm{r}}{\rm{i}}{\rm{b}}{\rm{u}}{\rm{t}}{\rm{e}}{\rm{d}}\,{\rm{l}}{\rm{o}}{\rm{a}}{\rm{d}}{\rm{i}}{\rm{n}}{\rm{g}}\,{\rm{o}}{\rm{n}}\,{\rm{t}}{\rm{h}}{\rm{e}}\,{\rm{s}}{\rm{u}}{\rm{r}}{\rm{f}}{\rm{a}}{\rm{c}}{\rm{e}}\,{\rm{o}}{\rm{f}}\,{\rm{t}}{\rm{h}}{\rm{e}}\,{\rm{V}}{\rm{C}}{\rm{s}})\end{array}$$

where **n** is the outward normal to the boundary. Number and range of static negative pressure problems was consistent with the loading sequence applied to the real device: *p* = [0 ÷ −500] *mbar* by fixed decremental steps of 50 *mbar*.

### Device Fabrication

Master molds of the two halves of the chip (representing the upper and lower microchannel layers) were fabricated in SU8-2075 negative photoresist (Microchem, Newton, MA) on 4″ silicon wafers following a standard photolithographic process according to the manufacturer’s protocols. Masters were silanized overnight in a chamber saturated with trimethylchlorosilane (Sigma-Aldrich, St.Louis, MO) vapors to facilitate demolding. The two halves of the chip were individually prepared by casting PDMS (Sylgard 184, Dow Corning, Midland, MI) at 10:1 v/v pre-polymer to catalyst ratio on the microfabricated mold, followed by thermal curing (1.5 h at 110 °C). Once cured, replicas were carefully peeled off from the mold, and vacuum/fluidic inlets and outlets were created using a suite of biopsy punches.

**Figure 7:**
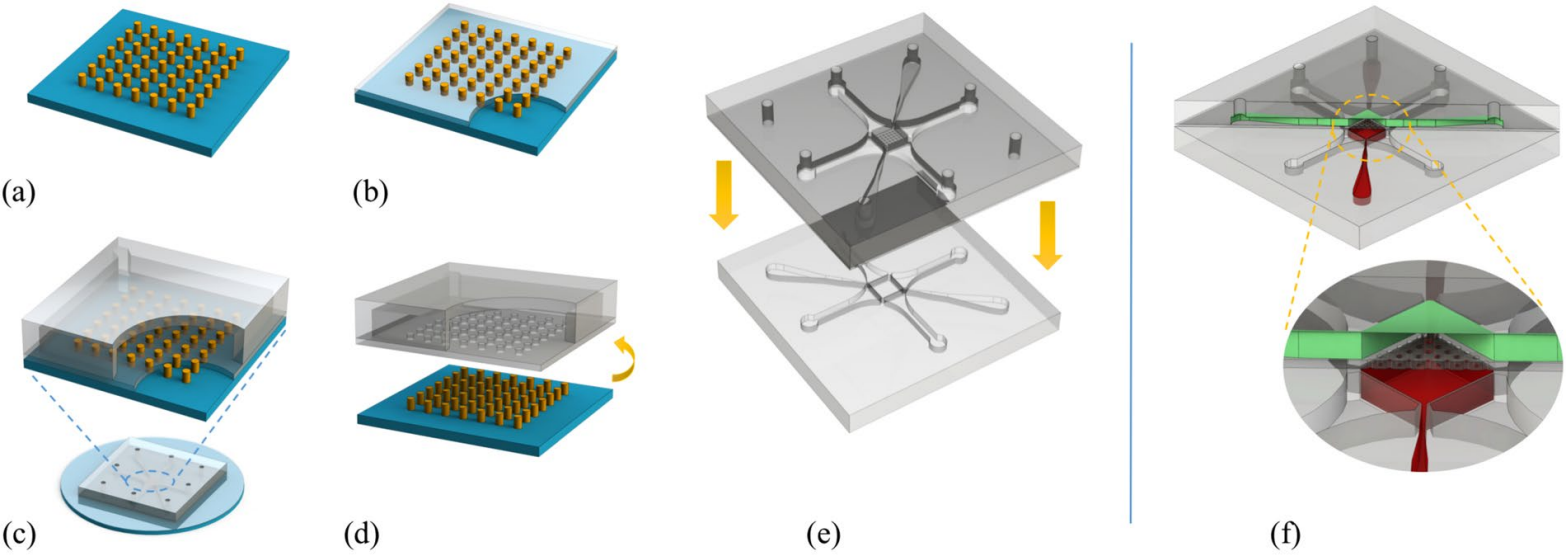
MCD microfabrication. Schematic representation of the membrane fabrication steps (**a,b**) and of the multi-step bonding process: upper half with the PM (**c,d**) and final alignment of the two halves (**e**). 3D schematic view of the assembled MCD (**f**): upper (green) and lower (red) culture chambers with the interposed PM (gray).

The PM was prepared by spinning a thin layer of PDMS (15:1 v/v) onto a photolithographically obtained SU8-on-silicon master (SU8-2050 negative photoresist, Microchem) containing an array of circular pillars (8 *μm* diameter × 45 *μm* height, with 55 *μm* spacing), followed by thermal curing for 3.5 h at 60 °C (Fig. 7(a) and (b)). Spin coating parameters were carefully optimized in order to obtain a 10-*μm* - thick membrane with circlewise through-holes. The surfaces of the PM (still on the master) and the upper microchannel layer were plasma-treated for 35 s at 20 *W*, immediately placed in conformal contact and further cured at 65 °C for 2 h (Fig. 7(c)). The assembly was then peeled off from the underlying master (Fig. 7(d)) and the portions of the membrane located over the VCs were torn off using forceps. Finally, the top microchannel layer (featuring the PM) and the bottom one were plasma-treated, carefully aligned (Fig. 7(e)) using a mask aligner (model MG1410, SET Corporation SA, St. Jeoire, France, purposely modified to accommodate the thickness of the substrates) and cured at 65 °C for 2 h. A schematic representation of the obtained assembly is presented in Fig. 7(f).

### Device Actuation

Actuation of the device was performed by applying controlled vacuum levels (in the 0 ÷ −500 *mbar* range at 50 *mbar* steps) at each actuator inlet using a multichannel programmable pressure controller (Elveflow OB-1 MK3, Elvesys, Paris, France). Membrane stretching was observed under a fully motorized inverted optical microscope (Eclipse Ti-E, Nikon Instruments, Tokyo, Japan) equipped with a high-sensitivity camera (Neo 5.5, Andor Technology, Belfast, UK) and a dedicated control software (NIS Elements AR, Nikon). In order to validate the *in silico* model, uniaxial and equibiaxial loading patterns were considered. Displacement field was calculated by tracking the displacement of PM pores using an image analysis algorithm (2D Object Tracking, NIS Elements) on the micrograph sequences at different pressure actuation levels. Device actuation is provided as Supplementary video S1.

**Table 1:** Fitting parameters of the three constitutive laws (7), (8), (9) adopted to model PDMS at 10:1 and 15:1 v/v pre-polymer to catalyst ratio. Nonlinear elastic material parameters (*a*, *b*, *c*) were obtained via Matlab routines with 95% confidence. Hyperelastic Mooney-Rivlin (*c*_1_, *c*_2_) and Ogden (*μ*, *α*) material parameters were obtained via least-square algorithms.

	Parameter	PDMS 10:1	PDMS 15:1	Unit
NLE	*a*	1.661 · 10^5^	1.494 · 10^5^	[Pa]
	*b*	2.366	0.726	[−]
	*c*	4.608	3.252	[−]
MR	*c*_1_	3.893 · 10^5^	8.083 · 10^4^	[Pa]
	*c*_2_	−9.976 · 10^4^	1.109 · 10^4^	[Pa]
OGD	*μ*	2.289 · 10^5^	2.752 · 10^5^	[Pa]
	*α*	3.717	1.417	[−]

## Electronic supplementary material

Supplementary information

Supplementary video
