# Supplementary material for: Computationally Informed Design of a Multi-Axial Actuated Microfluidic Chip Device
Source: Sci Rep. 2017 Jul 14;7:5489. doi: 10.1038/s41598-017-05237-9 (PMC5511244; doi:10.1038/s41598-017-05237-9)
Supplement: Supplementary file 1 — Supplementary information [file 41598_2017_5237_MOESM1_ESM.pdf]

SUPPLEMENTARY INFORMATION

Computationally Informed Design of a Multi-Axial Actuated Microfluidic Chip Device

Alessio Gizzi,[a] Sara Maria Giannitelli,[a] Marcella Trombetta,[a] Christian Cherubini,[a,b] Simonetta Filippi,[a,b] Adele De Ninno,[c] Luca Businaro,[c] Annamaria Gerardino,[c] and Alberto Rainer*[a,c]

PDMS mechanical characterization

Customized molds were purposely manufactured to investigate the mechanical response of the PDMS both in tension and compression. The degassed mixtures (10:1 v/v and 15:1 v/v prepolymer-to-curing agent ratio) were poured into the molds and cured using a digitally controlled oven, allowing precise control of the operating temperature. Since the influence of curing process on PDMS mechanical properties is clearly documented[1,2], curing time and temperatures were set in order to exactly reproduce the thermal steps experienced by the material during device fabrication.

Mechanical tests were performed on a universal tester (model 3365, Instron Corporation, Issaquah, WA, USA) equipped with a 500 N f.s. load cell. Unconfined compression tests were performed on cylindrical samples up to 50% strain. Uniaxial tensile tests were conducted until failure on rectangular specimens. For each experimental condition, at least three specimens were tested, stress–strain curves were acquired and averaged, and results were used for tuning of the *in silico* model.

Definition of the PM geometry

Different geometries were analyzed to identify the optimal MCD design (see Fig. S1). In Fig. S2, a direct comparison among three representative geometries under equibixial stretching is provided: displacement field (a), strain (b) and stress (c) patterns, equivalent stiffness (d). Color codes are homogenized over the three configurations.

Addendum to: Multiaxial Loading Simulations

Figure S3 shows von Mises strain field (a) and equivalent stiffness (b) induced on the optimized porous membrane (PM) under uniaxial (left), equibiaxial (center) and biaxial 3:5 (right) loading patterns for a maximum pressure $p = -500\,mbar$. Figure S4 compares the equibiaxial displacement field components (u, v horizontal and vertical, respectively) measured on the MCD with those obtained by numerical simulations for the three material models (NLE, MR, OGD). Three different points placed at $(0^\circ, 45^\circ, 90^\circ)$ along a circular region with radius 750 μm from the center of the PM have been represented.

References

1 D. T. Eddington, W. C. Crone and D. J. Beebe, 7th International Conference on Miniaturized Chemical and Biochemical Analysis Systems, 2003.

2 I. D. Johnston and et al., *Journal of Micromechanics and Microengineering*, 2014, **24**, 035017.

[a] *Department of Engineering, Università Campus Bio-Medico di Roma, Rome, Italy. E-mail: a.rainer@unicampus.it*

[b] *International Center for Relativistic Astrophysics (ICRA), Rome, Italy.*

[c] *Institute for Photonics and Nanotechnology, National Research Council, Rome, Italy.*

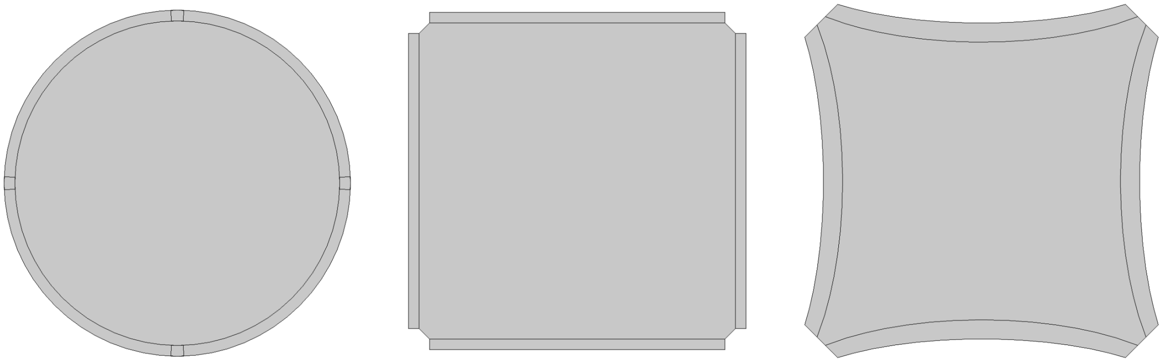

(a)

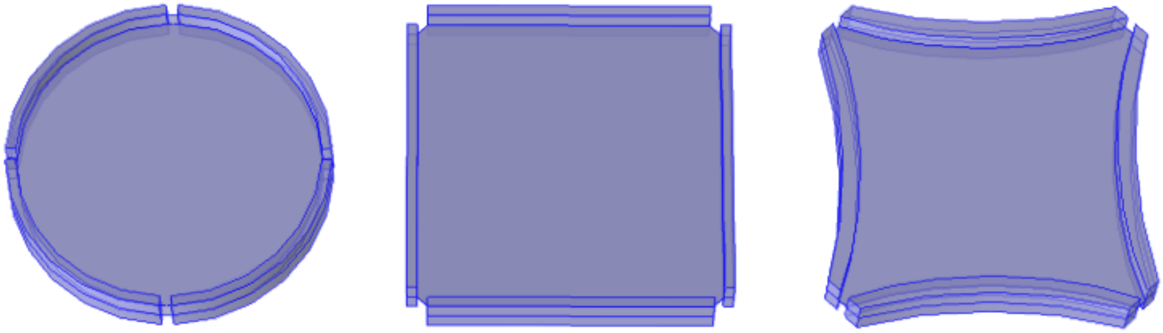

(b)

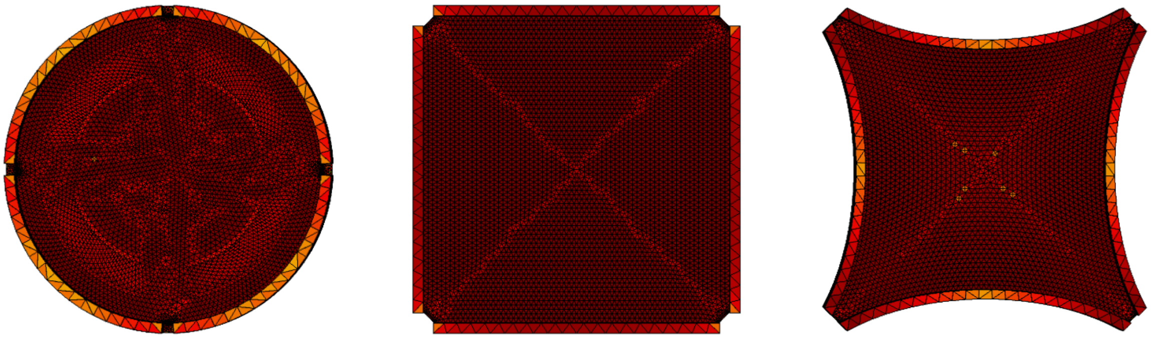

(c)

Fig. S1 Comparison of different designs for the PM: a) planar section; b) three-dimensional view; c) mesh quality.

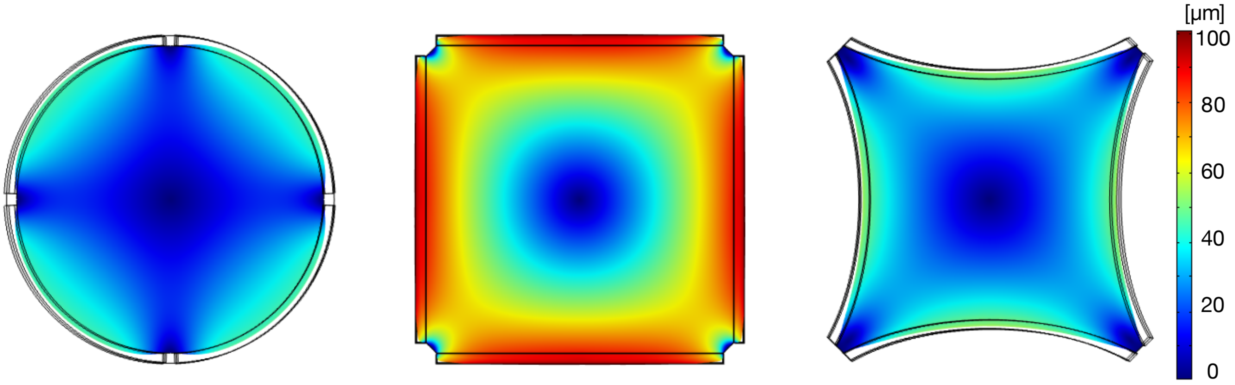

(a)

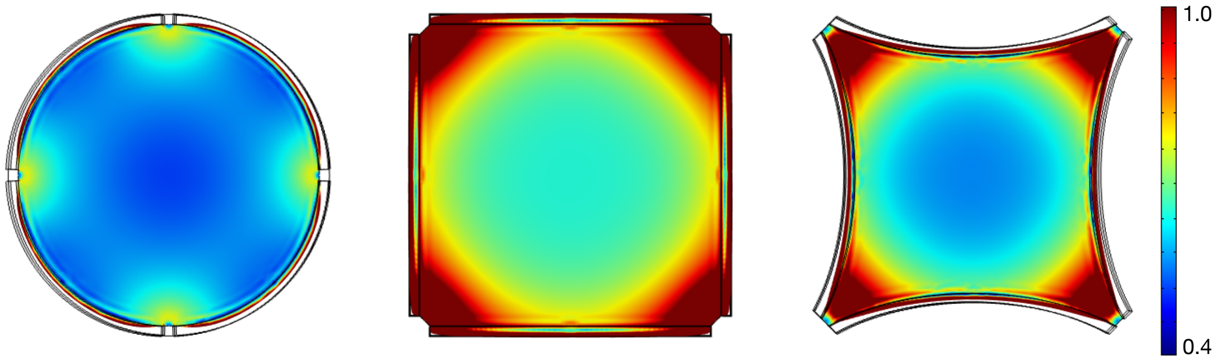

(b)

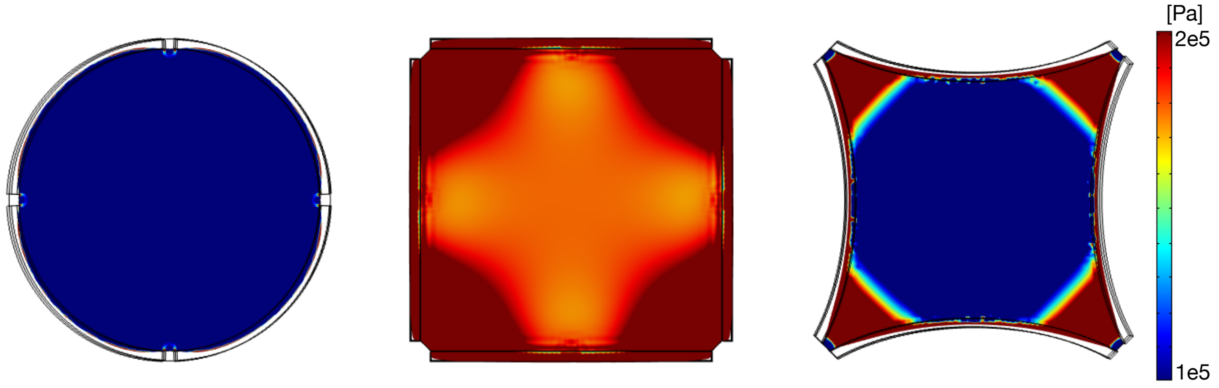

(c)

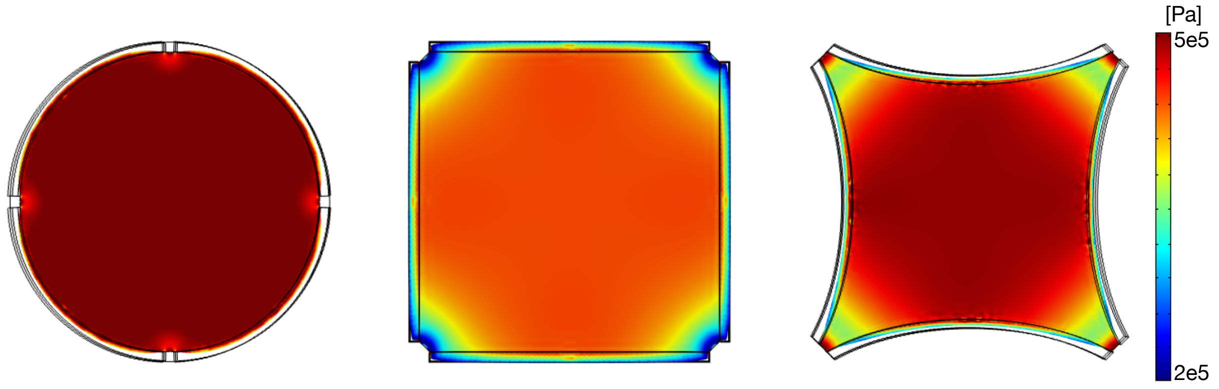

(d)

Fig. S2 Comparison of different designs for the PM under equibiaxial loading in terms of: a) displacement field; b) strain distribution of the first invariant of deformation; c) von Mises stress; d) equivalent stiffness.

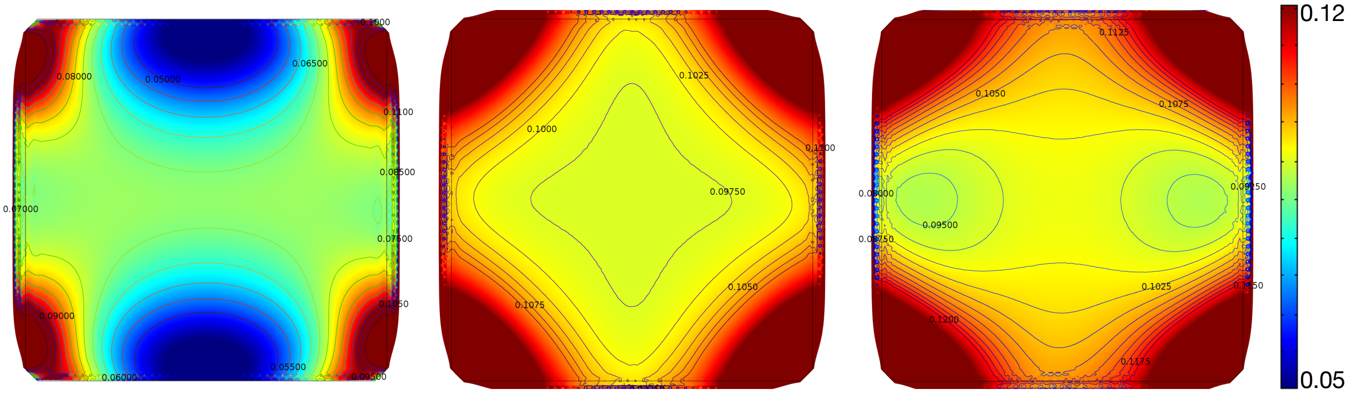

(a)

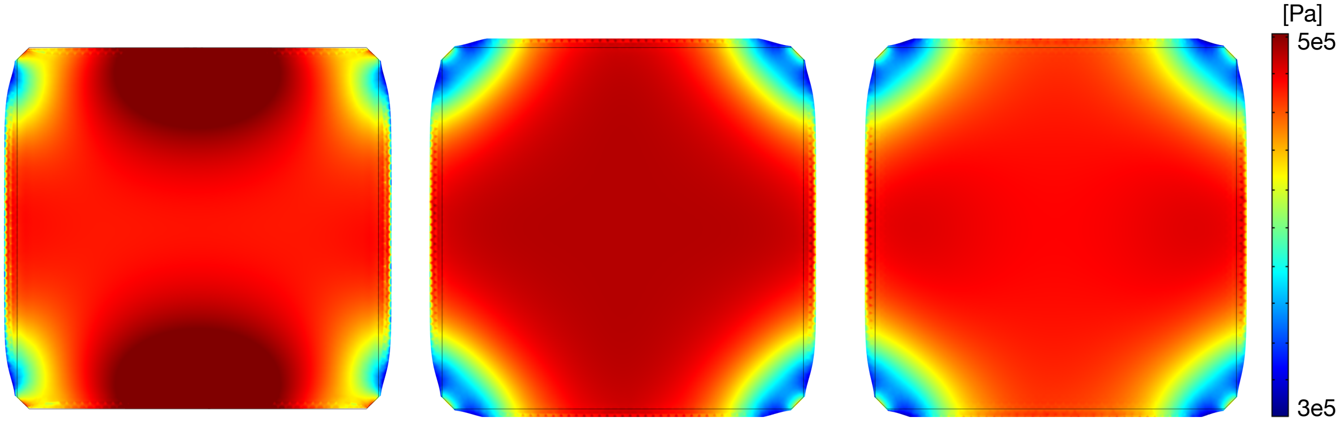

(b)

Fig. S3 a) von Mises strain induced on the PM under uniaxial (left), equibiaxial (center) and biaxial 3:5 (right) loading patterns for a maximum pressure $p = -500\ mbar$. A limited range of strain values $[0.05 \div 0.12]$ is displayed. b) Equivalent stiffness for the corresponding loading patterns. A limited range of stiffness values ($[3 \div 5 \cdot 10^5]$ Pa) is displayed.

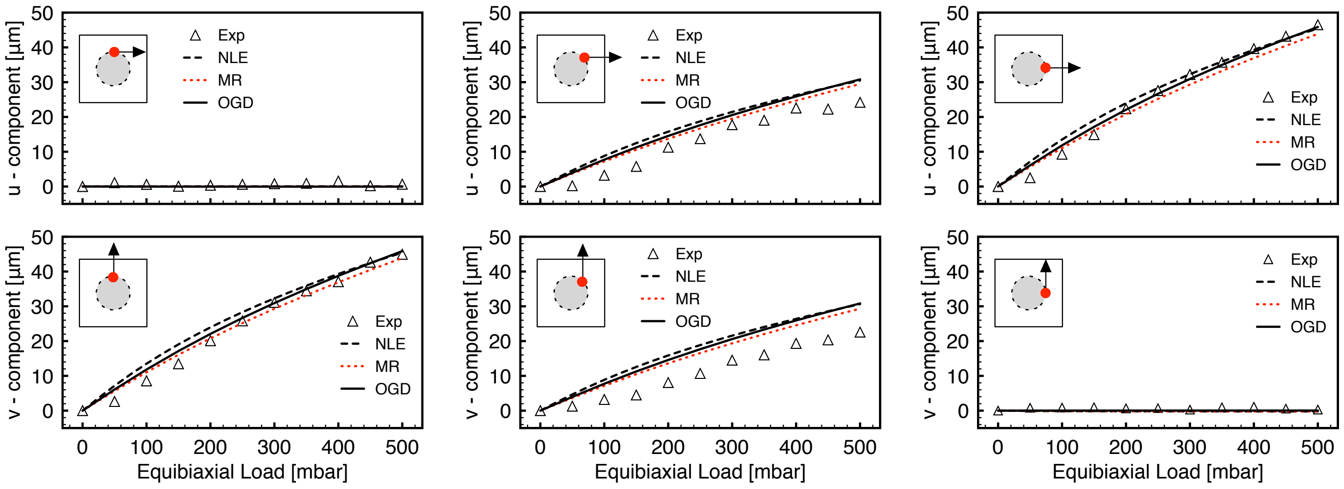

(a)

(b)

(c)

Radius [μm]	r.m.s. error [%]		
	NLE	MR	OGD
750	10.8	10.8	11.2

Fig. S4 Model validation under equibiaxial loading (negative pressure). Displacement field components (horizontal and vertical, u, v, respectively) taken at $r = 750\,\mu m$ (a-c) from the center of the porous membrane (PM) for three representative points. 'Exp' refers to measured data; NLE, MR and OGD refer to nonlinear elastic, Moonery-Rivlin and Ogden material models, respectively. The insets indicate the position of the points with coordinates (origin is set in the center of the membrane): (a) $(0, 750)$, (b) $(530, 530)$, (c) $(750, 0)$. The table reports the average percentage error of the displacement over the three selected locations for the peak pressure $(500\,mbar)$ vs. the three material models.
